# Healthier choices in an Australian health service: a pre-post audit of an intervention to improve the nutritional value of foods and drinks in vending machines and food outlets

**DOI:** 10.1186/1472-6963-13-492

**Published:** 2013-11-25

**Authors:** Colin Bell, Nicole Pond, Lynda Davies, Jeryl Lynn Francis, Elizabeth Campbell, John Wiggers

**Affiliations:** 1 School of Medicine and Public Health, University of Newcastle, Newcastle, Australia; 2 Hunter New England Population Health, Locked Bag 10, Wallsend, NSW 2287, Australia

**Keywords:** Vending machines, Food outlets, Intervention, Health care facilities, Healthy food and beverage availability

## Abstract

**Background:**

Vending machines and shops located within health care facilities are a source of food and drinks for staff, visitors and outpatients and they have the potential to promote healthy food and drink choices. This paper describes perceptions of parents and managers of health-service located food outlets towards the availability and labelling of healthier food options and the food and drinks offered for sale in health care facilities in Australia. It also describes the impact of an intervention to improve availability and labelling of healthier foods and drinks for sale.

**Methods:**

Parents (n = 168) and food outlet managers (n = 17) were surveyed. Food and drinks for sale in health-service operated food outlets (n = 5) and vending machines (n = 90) in health care facilities in the Hunter New England region of NSW were audited pre (2007) and post (2010/11) the introduction of policy and associated support to increase the availability of healthier choices. A traffic light system was used to classify foods from least (red) to most healthy choices (green).

**Results:**

Almost all (95%) parents and most (65%) food outlet managers thought food outlets on health service sites should have signs clearly showing healthy choices. Parents (90%) also thought all food outlets on health service sites should provide mostly healthy items compared to 47% of managers. The proportion of healthier beverage slots in vending machines increased from 29% to 51% at follow-up and the proportion of machines that labelled healthier drinks increased from 0 to 26%. No outlets labelled healthier items at baseline compared to 4 out of 5 after the intervention. No changes were observed in the availability or labelling of healthier food in vending machines or the availability of healthier food or drinks in food outlets.

**Conclusions:**

Baseline availability and labelling of healthier food and beverage choices for sale in health care facilities was poor in spite of the support of parents and outlet managers for such initiatives. The intervention encouraged improvements in the availability and labelling of healthier drinks but not foods in vending machines.

## Background

Health services have the potential to model a healthy environment through the provision of nutritious food and drink options for sale to staff, visitors and outpatients [1]. Health professionals routinely provide advice to clients on healthy eating and it is important that this advice is not undermined by lack of healthy food and drink items offered for sale in the health service environment. The limited evidence available however, is mostly from the U.S. and it suggests that the majority of foods available from outlets in health care facilities are unhealthy [2,3]. Furthermore, fast food outlets are common in hospitals [4]. Anecdotal evidence indicates the situation is similar in health services in Australia.

Improvements in the nutritional quality of foods and drinks available in hospitals are possible. A recent intervention in two New Zealand hospitals found that the introduction of nutrition criteria for vending machines did not affect sales volumes, led to increased staff satisfaction with vending products and, importantly, resulted in a substantial reduction in the amount of energy (-24%), total fat (-32%), saturated fat (-41%), and total sugars (-30%) per 100 g of product sold [5].

Because unhealthy diets are likely to be a major contributor to the burden of chronic disease in Australia [6], and in line with the national preventative health strategy [7], several states have introduced policy requiring public health sites to provide healthier food and drink choices from vending machines and retail outlets and to restrict unhealthy products [8-12]. NSW Health released their policy in late 2007. The policy uses a ‘traffic light system’ that classifies items using the colours red, amber and green, to indicate the least to most healthy choices, respectively. Good for Kids, Good for Life (hereafter Good for Kids) worked with health service management to introduce and implement this policy within the Hunter New England Local Health District (HNELHD) as part of wider efforts to provide healthier environments for children and their families. Good for Kids was a large multi-setting, multi-strategy childhood obesity prevention program run in the Hunter New England (HNE) region of NSW (2006-2010). To inform policy implementation we were also interested in the perceptions of health service clients (in this case parents) and providers (food outlet managers) on the availability and labelling of healthier choices within health service outlets.

The aims of this paper are to (1) report the perceptions of parents and managers of health-service located food outlets towards the availability and labelling of healthier food and drink options; (2) describe food and drinks available for sale from vending machines and food outlets in the HNELHD and (3); describe the impact of a policy-support intervention designed to increase the availability of healthier food and drink options and ensure they are labelled as such.

## Methods

### Design

A pre-post non-controlled study design was used to evaluate the intervention, which was implemented between 2008 and 2010. Telephone interviews and pen and paper surveys were conducted in 2007 with parents and food outlet managers respectively. Cross-sectional audits of vending machines and outlets selling food and drink were undertaken prior to implementation of the intervention (2007 for vending, 2008 for outlets). Repeat cross-sectional audits were undertaken in 2011 for vending machines, and in 2010 for outlets. The research was in compliance with the Helsinki Declaration and ethics approval was obtained from the Aboriginal Health and Medical Research Council (637/08) and the HNE Human Research Ethics Committee (HNEHREC 06/07/26/4.04).

### Context

The HNELHD employs approximately 14,500 staff and has many thousands of visitors and outpatients per annum to its health care facilities. Clinical facilities include 40 hospitals and 57 community health centres serving the whole population.

### Sample

The parent sample were HNE parents of children aged 2-15 years who completed a random digit dial telephone survey as part of Good for Kids (n = 168 drawn from a total sample of 941). The sample frame for the manager survey was a manager from all outlets selling food and drinks on HNELHD sites. In 2008 there were 19 food outlets on 10 sites. Five outlets were operated by the health service (four staff cafeterias and one kiosk), eight were fundraising kiosks operated by volunteers, and six were outlets under private contract. Only outlets operated by HNELHD staff were included in the follow-up audit as they were the only outlets to receive the full intervention (n = 5). In 2007 there were 112 vending machines on HNELHD sites, excluding those selling only hot drinks. The machines were located on 30 sites, with between one and 31 machines per site. Five sites had five or more machines. At follow-up in early 2011, there were 114 machines located on 30 sites, excluding those selling only hot drinks.

### Intervention

The intervention (Healthier Choices) was developed to be supportive of the NSW Health policy directive (first released in 2007, [6] amended in 2009, [7]). The policy directive addressed the provision of drinks and commercial ready-to-eat or pre-packaged foods (including salads and sandwiches) for vending machines and outlets selling food and drink. Standards around the provision of other types of food and drinks (those that require cooking or assembly other than salads and sandwiches) were to be addressed in future revisions of the policy directive. Nutritional standards used in the policy were based on an adapted version of the 'traffic light' nutrition classification system used as part of the NSW Fresh Tastes @ School Canteen Strategy [13]. The standards outlined in the policy directive included: to offer at least 80% healthier (green or amber) drinks and commercial ready-to-eat food items; to restrict serving sizes of red (least healthy) drinks to 375 ml or less and; to label healthier options. Prior to the intervention, no specific requirements were in place to improve the nutritional value of food and drinks sold in vending machines or outlets. The policy directive did not apply to hospital inpatient menus for which specific nutritional guidelines already existed. Table 1 shows strategies implemented as part of the Healthier Choices program. Although not available at the time of this intervention, the nutritional criteria and standards used were similar to those that can be found at http://www.health.nsw.gov.au/pubs/2010/pdf/llw_user_guide.pdf. Development and delivery of the intervention was led by health service staff and the health service contracts manager (for vending) under the guidance of an advisory committee and with support from the health service executive. For vending machines, requirements were incorporated into the tender process for a five-year contract signed in late 2008. The intervention was offered to all outlets selling food and drinks on health service sites (n = 19). However, the private outlets (n = 6) were exempt from compliance with the NSW policy directive because pre-existing contracts did not require it and fundraising outlets (n = 8) became exempt with the re-release of the policy in 2009 and therefore did not receive the full intervention.

**Table 1:** Strategies to improve the nutritional quality of foods in vending machines and food outlets in the Hunter New England Local Health District (HNELHD)

**Component**	**Strategies implemented**	
	**Vending machines**	**Food outlets**
**Building leadership and consensus**	LHD Advisory Committee	LHD Advisory Committee
	Memos from HNELHD executive to site managers to encourage support	Memos from HNELHD executive to site managers to encourage support
	Engagement of HNELHD contracts manager	
**Resources, tools, information; incorporation into systems/procedures**	Development of HNELHD Vending Policy Compliance Procedure*	Development of HNELHD Outlets Policy Compliance Procedure*
	*Healthier Choices* requirements built into tender and contract processes for supply of vending machine services	*Healthier Choices* Guide and resources disseminated during site visits: logo and signage for products; posters; classification of product table; taste testing kit;% calculation tool
	HNELHD vending contract Nov 2008 included *Healthier Choices* conditions	
	Contractor provided with: *Healthier Choices* logo for vending machines; and classification system resource	*Healthier Choices* fact sheets circulated to outlets each year
		Offer of revised menu board with green and amber items labelled
**Training**		Invitation to outlet managers to attend Healthy Canteen expo
**Follow up support**	Reactive- dietitian advice on request†	Proactive - dietitian support
		Site visits - two per year
		Telephone support calls
		Reactive – dietitian advice available on request via email or phone
**Monitoring and feedback**	Reactive feedback to contractor on planograms for planned stock†	Audit monitoring and feedback - tailored written reports to outlet managers

*The Policy Compliance Procedure outlined that requirements be built into tender or contract processes. There was minimal capacity for this for outlets within this intervention period; however *Healthier Choices* requirements were built into tender and contract processes for one private outlet contract enacted during 2010. This outlet is not included in this evaluation as it had not been set up at follow up audit. Policy Compliance Procedures also included a communication strategy that included presentations to key stakeholders including health service managers and dieticians.

†Feedback provided twice for planned drinks machines and once for planned snack machines, other advice on *Healthier Choices* was not sought.

### Data collection and measures

#### ***Perceptions of healthy food availability and labelling in health services***

Parents and food outlet managers were asked if food outlets on health service sites should provide mostly healthy items, have signs clearly showing healthy choices, restrict the sale of unhealthy food or drink, ban the sale of unhealthy food or drinks (parents), or not sell any red products within two years (managers). Participants responded to statements (see Table 2 for wording) using a 5-point likert scale from strongly agree to strongly disagree.

**Table 2:** Parents (n=168) and food outlet (n=17) manager’s perceptions (2007) about availability of healthy foods in health-service food outlets

**Item**	**Parents who agree or strongly agree, n(%)**	**Managers who agree or strongly agree, n(%)**
All food/drink outlets on health service sites should provide mostly healthy items	151(90)	8(47)
My outlet should provide mostly healthy choices	-	7(41)
All food/drink outlets on health service sites should have signs clearly showing healthy choices	160(95)	11(65)
Food/drink outlets on health service sites should restrict the sale of unhealthy food/drink	139(83)	4(24)
Health services should ban the sale of unhealthy food/drink from health service site outlets	67(40)	-
Food outlets on health sites should not sell any red products within 2 years	-	2(12)

### Availability of healthier foods/drinks and labels

Information on products offered in each slot of vending machines (including brand, size, flavour), and on all items visible for sale or listed on menu boards in outlets, was collected by project staff on standardised audit forms and classified into product types. Included in the tally of commercial ready-to-eat foods were those in packages, salads and sandwiches and foods eaten in the form they are received by the outlet (they may need toasting or re-heating) but required no further preparation or cooking. The presence of signage or labels distinguishing healthier choices was also recorded. Labelling on vending machines was a coloured dot on the slots, plus a colour code guide on the machine. Outlets had coloured dots and/or basket shelf markers with 'healthier choices' with some explanatory signage for the colour coding. Product types were classified as red, amber or green by a dietitian (NP) using an expanded version of the NSW Health criteria [8], drawing on criteria used in NSW schools from the Healthy Kids Canteen Association [13].

### Analysis

Analysis was undertaken in Excel and SAS version 9.2. Baseline perceptions of parents and food outlet managers were summarised using descriptive statistics. For vending machines, baseline and follow-up data were treated as independent cross sectional samples and the main outcome was differences in the percentage of slots (excluding empty slots) in each machine classified as either amber or green (amber/green). Differences were tested separately for drinks and foods/snacks using non-parametric Kruskal-Wallis tests. For outlets, analysis was restricted to the five outlets operated by the local health district. The primary outcome was differences between baseline and follow-up in the percentage of items classified as amber/green offered by each outlet, calculated separately for drinks and foods/snacks and tested using paired t-tests. Labelling was defined as ‘at least one green or amber product accurately labelled’. The tests had the power to detect mean changes of ≥ 20% as statistically significant (standard deviation = 16, alpha =0.05, power =80%).

Fishers exact tests were used to compare differences between baseline and follow-up in secondary outcome measures: vending machines meeting 80% amber/green standard (yes, no); vending machines with all red drinks 375mls or less (yes, no), and machines labelling green/amber items (yes, no). Vending machines were considered appropriately labelled if all green and amber products were accurately labelled and information was provided on the machine explaining the labels.

## Results

### Parent and manager surveys

Table 2 summarises parents’ and managers’ perceptions on the availability of healthier choices in health service food outlets.

The response rate of parents for the CATI interview was 63%. Parents were predominantly female (83%) with a mean age of 39 years. Twenty-three percent had a university education. Almost all (90%) of the parents surveyed thought that outlets should sell mostly healthy food items. Most parents (95%) thought that healthy choices should be clearly labelled and 83% thought health services should restrict the sale of unhealthy food and drink. Forty percent were in favour of a ban on unhealthy products and only a minority strongly disagreed with such a ban (9%, not shown).

Seventeen of 19 (89%) food outlet managers completed the perceptions survey. Of these, seven were classified as fundraising and four were exempt from the policy as they remained within a retail lease arrangement in a major hospital over the intervention period. Unlike parents, less than half (47%) of the food outlet managers surveyed thought health-service based outlets should provide mostly healthy items and even fewer (41%) thought their own outlet should. Very few (24% and 12% respectively) were in favour of restrictions on the sale of unhealthy food/drink or a 2-year phase out of red products. However, the majority (65%) of managers supported promotion of healthy items through signage.

### Audits

#### ***Vending machines***

The baseline audit was conducted on 88 vending machines (79% of machines) located on 24 sites. The follow-up sample consisted of 90 machines (80% of machines) located on 26 sites. Table 3 provides information on the proportion of green/amber products sold in HNELHD vending machines. At baseline, the mean proportion of amber/green drinks and foods per machine were 29% and 1% respectively and no labelling was evident. The mean proportion of amber/green drinks at follow-up (51%) was significantly higher (p<0.05) than at baseline (29%). Very few machines (four) met the 80% standard at follow up and there was no significant difference with baseline (p>0.05). The proportion of machines that met serve size restriction standards for all red drinks increased from 31% to 44% but remained under half of machines and again the increase was not statistically significant. Machines typically included sports drinks, flavoured, sweetened waters and iced teas in 500 ml to 600 ml sizes. Labelling of healthy options did increase from 0% to 26% of machines selling drinks at follow-up (p<0.01), but with respect to machines selling food, the follow-up values for each outcome were not significantly different to baseline.

**Table 3:** Nutritional quality of food and drinks in vending machines at baseline and follow-up

**Variable**	** Machines selling drinks**^**a**^		** Machines selling food**^**a**^	
	**2007 (n=61)**	**2011 (n=62)**	**2007 (n=34)**	**2011 (n=47)**
Amber/green slots per machine, mean %	29%	51%*	1%	3%
Machines with at least 80% amber/green, n (%)	0	4 (6%)	0	1^b^ (2%)
Machines with all red drinks 375 ml or less, n (%)	19^c^ (31%)	27 (44%)		
Machines with amber/green items labelled, n (%)	0	16 (26%)**	0	3 (6%)

*p<0.05, **p<0.01.

^a^Combination machines selling both drinks and snacks are included in totals. The number of combination machines at baseline was 7 and at follow-up was 19.

^b^This was a sandwich machine.

^c^These machines were selling cans of drink only.

#### Outlets

At baseline, only 59% of drinks and 41% of foods available in all 19 outlets were classified as green or amber. Compared to other categories (savoury snack foods and crisps = 19%, deserts, sweet baked goods and ice-creams = 35%, and meat items, hot foods, sandwiches and salads = 87%), sweet snack foods, bars and confectionary was the food category that had the lowest proportion of green/amber foods (9%). We also found that fundraising outlets (37%) had less green/amber foods and drinks available than privately operated (43%) or health service operated (60%) outlets.

Results for the five outlets operated by HNELHD assessed at baseline and follow-up are shown in Table 4. There was an increase in the percentage of amber/green drinks (58% to 72%, non-significant) and foods (60% to 69%, non-significant) available. However, only two of five outlets met standards for 80% green/amber drinks and foods at follow-up. Three of the five outlets met standards for red drink serve size restrictions. Labelling of healthier food and drink options occurred at four outlets at follow-up, compared to no labelling at baseline.

**Table 4:** Nutritional quality of food and drinks in health service operated food outlets at baseline and follow-up

**Variable**	**Year**	
	**2008 (n=5)**	**2010 (n=5)**
Amber/green drinks per outlet, %	58%	72%
Outlets with at least 80% amber/green drinks, n	0	2
Outlets with all red drinks 375 ml or less, n	1	3
Amber/green foods per outlet, %	60%	69%
Outlets with at least 80% amber/green foods, n	1	2
Amber/green food and drink options labelled	0	4

## Discussion

An audit of vending machines and food outlets in HNELHD sites found that healthier (green/amber) food and beverage choices were not readily available. Only 29% of beverages available in vending machines were healthier choices and almost none of the foods (1%). Moreover, foods and beverages were poorly labelled in spite of strong support from parents for the provision and labelling of healthy food and drink choices in health facilities. The intervention significantly improved the availability and labelling of healthier drink choices in vending machines. However, it did not improve the availability of healthier foods in vending machines and it did not have a major influence on the availability of healthy choices in food outlets.

We are aware of one other Australian study that has measured the impact of a healthier choices strategy in health care facilities. The Queensland Government introduced the ‘A Better Choice Healthy Food and Drink Supply Strategy’ for Queensland Health Facilities in September 2007. The aim was to increase the supply of healthy food and drink to Queensland Health staff, visitors and the general public in Queensland Health facilities. The strategy became mandatory on 1 September 2008 and it was evaluated in 2009 [9]. Most of the 278 facilities surveyed (78 percent) reported implementation of more than half of the requirements of the strategy, and 25 percent reported full strategy implementation. Reports from a majority of facilities indicated that 82% of food outlets had restricted the supply of red category foods and drinks to <20% of items on display. Also, almost three-quarters (74 per cent) of facilities reported that red category food and drinks had been completely removed from vending machines. However, consistent with our finding that changes to food/snack items stocked in machines proved harder than changing drinks, the Queensland study reported that changing snack vending machines was difficult [9]. This may be because it is easier to source healthier drink alternatives, such as bottled water and low fat milks, than source appropriate healthier food alternatives that have a long shelf-life, are of appropriate size and shape (i.e. fit in the vending machine slots) and are comparatively priced.

Several factors may have contributed to the intensity and reach of our intervention being less than desired for both vending machines and food outlets. Intensity was limited by delays in finalising the vending contract and, once the contract was in place, there were additional delays on many sites related to machine installation and confusion over whether or not machines had to comply with the policy directive. The exemption for fundraising and existing contracts clearly limited the reach of the intervention. There were also logistical challenges reaching outlets. Distance and non-use of email made contact, training and follow-up support hard, particularly given that managers of these outlets were often volunteers and part-time. Also, performance monitoring and feedback may not have been sufficient to encourage compliance. More strategic use of the information on parent (customer) perceptions may have encouraged managers to supply foods and beverages more in line with demand and ideas for healthier fundraising alternatives may have led to better compliance from the fundraising outlets. For example, NSW schools have identified creative ways of making money that promote health and wellbeing such as selling fruit and vegetable boxes [13]. Being purposeful about promoting fruit and vegetables in health services may also be beneficial. In the US, regular farmer’s markets in Kaiser Permanente’s health facilities have led to improvements in the fruit and vegetable consumption of patrons [14].

Another barrier to increasing healthier choices may have been concerns about loss of profits. Indeed, this was the reason fundraising volunteer groups lobbied against the original policy directive at state level, leading to the exemption. In a survey of Canadian and US Paediatric hospitals, McDonald and colleagues found that revenue from food outlets, or the presence of more internally-operated cafeterias, was positively associated with availability of less nutritious foods and inversely associated with availability of healthful food and beverage alternatives [2]. This suggests that a reliance on or drive for revenue may promote the sale of less nutritious food. Thus, it may have been helpful if we had provided information and examples on how vending machines and food outlets can sell mostly healthy food and still turn a profit. Also, given the improvements in labelling and that 65% of outlet managers agreed that food outlets on health service sites should have signs clearly showing healthy choices, an easier first step may have been the introduction of shelf labels.

The strengths of this study include the use of audits rather than self-report for collecting data on food availability and working with dietitians to determine food and drink classifications against previously developed criteria. Also, we were able to quantify the level of parental support for making healthier choices available in health facilities. The main limitation was the absence of a comparison group, restricting our ability to attribute changes to the intervention. Small sample sizes precluded meaningful statistical analysis in some cases. Also, a one-off audit may not be representative of items typically offered for sale although, for vending, interim reports from the contractor were consistent with the audit findings.

Efforts to improve the health of foods available in health care facilities are not new in Australia. The New South Wales Health Department developed a nutrition strategy in 1995 to promote better nutrition for people in NSW [15], and in line with this strategy, a number of area health services, including Hunter, adopted food and nutrition policies [1]. These initiatives may have improved the nutritional quality of foods for patients [16,17], but it is evident from renewed efforts to introduce policy and the high prevalence of unhealthy ‘red’ foods found in this study that there is also a need to improve the nutritional quality of food available for sale to staff, visitors and outpatients.

## Conclusions

Our study has demonstrated that healthier choices are supported by senior health service managers and by parents and that the intervention helped with policy implementation by increasing the availability and labelling of healthier beverage choices in some outlets. However, existing contracts, business interests and exemptions hindered more widespread policy implementation and intervention effectiveness. We recommend the use of the strategies in Table 1 to support the implementation of healthy food and beverage policy in other health services. Healthier Choices policy should be applied to contracts as they come up for tender and creative strategies are needed to ensure fundraising activities promote health and well-being.

## Competing interests

The authors declare no financial or non-financial competing interests.

## Authors’ contributions

ACB, EC and JW conceived of the study, and participated in its design and coordination and helped to draft the manuscript; NP and LD participated in the design of the study, coordinated the intervention and helped to draft the manuscript. JLF performed the statistical analysis and helped to draft the manuscript. All authors read and approved the final manuscript.

## Pre-publication history

The pre-publication history for this paper can be accessed here:

http://www.biomedcentral.com/1472-6963/13/492/prepub
